# Perianal disease onset age is associated with distinct disease features and need for intestinal resection in perianal Crohn’s disease: a ten-year hospital-based observational study in China

**DOI:** 10.1186/s12876-021-01961-9

**Published:** 2021-10-14

**Authors:** Haichao Wang, Yaling Wu, Chen Ye, Zhanju Liu, Xiaolei Wang

**Affiliations:** 1 Department of Gastroenterology, Shanghai Tenth People’s Hospital, School of Medicine, Tongji University, Shanghai, 200072 China; 2 Medical College of Soochow University, Suzhou, 215000 Jiangsu Province China

**Keywords:** Perianal disease, Crohn’s disease, Paediatric onset, Adult-onset, Intestinal resection

## Abstract

**Background and aims:**

The significance of different ages of perianal disease (PD) onset in patients with perianal Crohn’s disease (PCD) remains unknown. We aimed to investigate the impact of paediatric-onset PD (POP) and adult-onset PD (AOP) on the Crohn’s disease (CD) course in a Chinese cohort.

**Methods:**

The medical records of diagnosed PCD patients from 2008 to 2018 were reviewed retrospectively. The cumulative incidence and predictors of intestinal resection were calculated using the Kaplan–Meier and logistic regression analysis.

**Results:**

Complex perianal fistulas (71.7% vs 50.0%, *p* = 0.011) and infliximab (IFX) treatment (33.3% vs 22.0%, *p* = 0.044) were more common among the POP patients (age < 18 years old, n = 84). A younger PD onset age (15.1 ± 2.9 vs 30.2 ± 10.5 years, *p* < 0.001) and shorter PCD diagnostic delay (12 vs 24 months, *p* = 0.033) was found in the POP cohort. AOP patients (age ≥ 18 years old, n = 209) had a higher rate of current smoking (12.9% vs 4.8%, *p* = 0.040), stricturing behaviour (42.1% vs 27.4%, *p* = 0.024) and intestinal resection (21.1% vs 4.8%, *p* = 0.001). The cumulative probability of intestinal resection in AOP patients was higher than that in POP patients (*p* = 0.007). In multivariable analysis, AOP (OR: 4.939, 95% CI 1.538–15.855, *p* = 0.007), stricturing behaviour (OR: 1.810, 95% CI 1.008–3.251, *p* = 0.047) and rectal inflammation (OR: 3.166, 95% CI 1.119–8.959, *p* = 0.030) were predictive factors for CD-related intestinal resection in all PCD patients. AOP patients with complex perianal fistula (OR: 2.257, 95% CI 1.041–4.891, *p* = 0.039) and POP patients with rectal inflammation (OR: 3.166, 95% CI 1.119–8.959, *p* = 0.030) were more likely to suffer intestinal resection. The IFX administration significantly decreased the rate of intestinal resection in AOP patients (*r* = − 0.900, *p* = 0.037).

**Conclusions:**

The AOP patients have more complicated luminal disease and higher rate of intestinal resection than COP patients. The perianal diseases onset-age can provide clinical treatment guidance for individual management of CD patients.

## Introduction

Crohn’s disease (CD) is a multifactorial systemic inflammatory bowel disease (IBD) with a heterogeneous clinical course. Perianal disease (PD) is a common complication in CD patients and can lead to a low quality of life, a high rate of surgical interventions, and challenging therapeutic situations [1–3]. Patients with perianal Crohn’s disease (PCD) undergo a number of perianal surgical interventions (PSIs), resulting in multiple disabilities. However, the successful development of various anti-tumour necrosis factor (TNF)-α agents (the first biological agents for CD in the world) breaks this impasse. Due to their rapid efficacy in cases of complicated PD and luminal lesions, anti-TNF agents, including infliximab (IFX) and adalimumab, are recommended as the first-line treatment for CD patients who have high-risk factors for poor outcomes, including PD, onset age < 40 years, and extensive small bowel involvement [4–10]. In China, IFX was the only available anti-TNF agent for CD from 2008 to 2018 and showed a more favourable perianal and luminal outcomes than traditional treatment in both children and adult PCD patients [11, 12]. There is some evidence that the prevalence of PCD was higher in Asian than Western countries, and the outcomes of Asian PCD patients may differ from those of Western patients [13–15]. However, Chinese studies evaluating the characteristics and outcomes of PCD patients are limited.

Patients with PCD usually have a higher risk of abdominal surgery. However, this increased risk only reached statistical significance in patients who developed PD during follow-up, but not in patients who presented with PD before or at luminal CD diagnosis [1]. This finding indicates that the PD onset time plays an important role in the CD natural history. Approximately 25% of CD patients are diagnosed in childhood or adolescence [16]. Interestingly, different features have been found between paediatric- and adult-onset CD patients. For example, paediatric CD patients had a three-times higher risk of progressing to complicated CD than adult-onset CD patients [17] and required more intensive pharmacological treatments [17–19]. However, Schoepfer, A., et al. reported that adult CD patients present more bowel surgery and longer diagnostic delay (the time interval between the first symptom and diagnosis of CD) compared with paediatric CD patients. And a long diagnostic delay was found to be predictive for an increased incidence of CD-related surgery only in the adult but not in the paediatric CD patients [18]. The diagnosis of perianal fistulas after the luminal CD was reported to range from 5 to 21% in adult CD patients and 8% to 27% in paediatric CD patients [4–7]. These suggest that onset-age plays an important role in the CD disease course. However, whether the onset-age of PD is associated with the incidence of CD-related intestinal resection is still unknown. Therefore, we aimed to evaluate the measurable differences in clinical outcomes between PCD patients with paediatric-onset PD (POP) and adult-onset PD (AOP) and to identify clinical predictive factors of the development of intestinal resection in this ten-year follow-up study.

## Materials and methods

### Patients

We retrospectively evaluated 747 consecutive CD patients in the Tenth People’s Hospital affiliated with Tongji University (Shanghai, China) from January 2008 to December 2017. Eligible patients were confirmed CD patients having PD involvement during the disease course. Both the PD presented before and after diagnosis of CD were included in the study. The diagnosis of CD in paediatric patients was based on the Porto criteria [20], and the diagnosis of CD in adult patients satisfied the criteria published by the European Crohn’s and Colitis Organization [21]. PD was diagnosed according to presence of perianal symptoms, results of clinical examinations, perianal ultrasound and/or magnetic resonance imaging. Patients who were lost to follow-up or had incomplete clinical information were excluded. The follow-up period refers to the period from the first hospitalization to the last hospitalization within the end of data collection (December 31, 2018). Patients were followed up at each visit in hospital according to the medical records, and occasional telephone consultation. The patients with follow-up period less than 3 months were excluded. The study protocol was reviewed and approved by the Institutional Ethics Committee of the Tenth People’s Hospital affiliated with Tongji University, Shanghai, China (SHSY- IEC- 4.0/19 -23/01), and got an exemption from informed consent because of the retrospective study design.

### Description of variables and outcomes

The medical records were reviewed for sex, PD onset age, PD type, age at CD diagnosis, age at PCD diagnosis, PCD diagnostic delay, clinical manifestations, Montreal classification, smoking status, family history of IBD, CD-related medications and surgical interventions (perianal and abdominal interventions). CD-related medical treatment included antibiotics (imidazole and quinolones), immunosuppressants (azathioprine and methotrexate), corticosteroids and IFX. IFX was regularly administered intravenously at a dose of 5 mg/kg body weight at 0, 2, and 6 weeks and then every 8 weeks. The duration of IFX treatment was calculated from the first infusion to the last infusion within the follow-up period. The PSIs included perianal abscess incision and drainage, seton insertion, fistulectomy, de-functioning surgery, and proctectomy. CD-related abdominal surgery in this study mainly referred to CD-related intestinal resection, including the partial ileal resection, partial colon resection, ileocolonic resection.

POP was defined as PD with an onset age of less than 18 (< 18) years, and AOP was defined as PD with an onset age of greater than or equal to 18 (≥ 18) years [18]. The types of PD were classified according to a technical review by the American Gastroenterology Association at the time of PD diagnosis and included perianal skin lesions (anal skin tags and haemorrhoids), anal canal lesions (anal fissures, ulcers, and anorectal strictures), perianal abscesses, perianal fistulas, rectovaginal fistulas, and cancer [22]. Perianal fistulas were classified into simple and complex fistulas. Simple perianal fistulas are low in position (superficial, low intersphincteric or low transsphincteric origin of the fistula tract) and have only a single external opening without evidence to suggest perianal abscesses, anorectal strictures or rectovaginal fistulas. Complex perianal fistulas originate from a high position (intersphincteric, transsphincteric, extrasphincteric, or suprasphincteric region) and usually have multiple external openings combined with perianal abscesses, anorectal structures, or rectovaginal fistulas [22]. PCD diagnostic delay is defined as the time interval between the first diagnosis of PD and luminal CD. The first diagnosis of PD depends on the exact medical records of the electronic system in our hospital or the accurate data records in other hospitals provided by patients themselves. The diagnosis of luminal CD is defined by the findings of endoscopy in our hospital or endoscopic report in the other hospitals.

The clinical outcomes of PD were categorized as clinical remission, non-remission and recurrence. Clinical remission was defined as follows: (1) no perianal symptoms (e.g., local swelling and pain, haematochezia, faecal incontinence); (2) closure of the external opening of perianal fistulas; (3) no visible internal openings, perianal abscesses or anal ulcers; and (4) absence of drainage despite gentle finger compression. Non-remission referred failure to achieve clinical remission after a certain therapeutic strategy or maintain clinical remission for less than 3 months. Recurrence was defined as the presence of new PD after 3 months of clinical remission.

### Statistical analyses

Categorical variables are described as counts with percentages and were assessed by the chi-square test or Fisher’s exact test. Continuous variables are described as the mean ± standard deviation (SD) or median plus interquartile range (IQR) and were analysed by the independent sample t-test or the Mann–Whitney test. The factors associated with CD-related surgery were analysed by univariate and multivariate analyses. Multivariate logistic regression analysis was performed for factors with a *p* value < 0.2 in the univariate analysis by the enter method (Wald test used for assessing *p* values). Kaplan–Meier analysis was applied to calculate the cumulative probabilities of IFX treatment and CD-related intestinal resection during the 10-year follow-up period. A log-rank comparison was used to compare the cumulative probabilities between the two groups of patients with POP and AOP. Spearman correlation coefficients were calculated between the rates of CD-related intestinal resection and the percentages of patients treated with IFX. Two-sided *p* values < 0.05 were considered statistically significant. SPSS 22.0 software (IBM, Sommers, NY, USA) was used for statistical analysis.

## Results

### Characteristics of PCD patients with POP and AOP

**Fig. 1:**
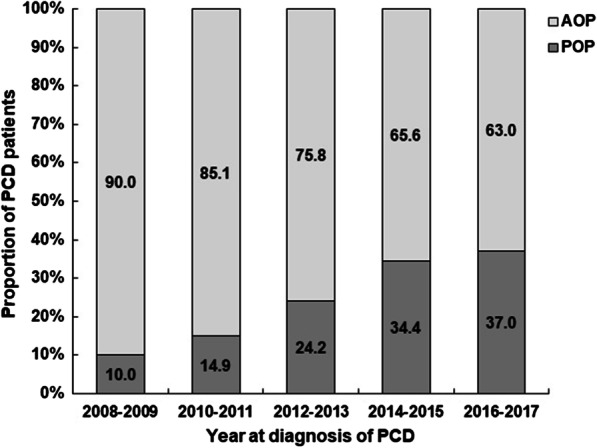
Proportion of PCD patients with different perianal disease onset ages between 2008 and 2017. *PCD* perianal Crohn’s disease, *POP* paediatric-onset perianal disease, *AOP* adult-onset perianal disease

Among 747 well-characterized CD patients, 293 (39.2%) patients with perianal involvement were enrolled. The median follow-up duration among the 293 PCD patients was 72 (IQR: 36–108) months. Two hundred and four (69.6%) patients had the one type of perianal diseases, 68 (23.2%) having two types of PD, and 17 (5.8%) patients having three and over three types of PD during the disease course. Perianal fistula was the most common PCD (163/293, 55.6%), with complex phenotypes in 57.1% (93/163) of cases. One hundred forty-one (48.1%) PCD patients had perianal abscesses, and 51 (17.4%) had haemorrhoids. Anal ulcers, fissures and rectovaginal fistula and were seen in only 5.1% (15/293), 4.4% (13/293) and 3.4% (10/293) of PCD patients, respectively. No cases of cancer were identified during follow-up. The mean onset age of PD was 25.9 ± 11.3 years old. The AOP cohort comprised 209 (71.3%) patients, and the POP cohort included 84 cases (28.7%). There was a gradual increase in the diagnosis of POP in PCD patients from 2008.1 to 2017.12 but a reduction in the diagnosis of patients with AOP during the same period (Fig. 1).

The baseline characteristics of these two groups of patients were summarized in Table 1. Most POP (81.0%) and AOP (73.7%) patients were male. Among POP patients, the mean age at diagnosis of CD (18.0 ± 4.3 vs 32.0 ± 10.9 years, *p* < 0.001) and at onset of PD (15.1 ± 2.9 vs 30.2 ± 10.5 years, *p* < 0.001) was significantly younger than that among AOP patients (Table 1). The median duration of PCD diagnostic delay (12 [IQR: 2–48] vs 24 [IQR: 2.5–60] months, *p* = 0.033) was shorter in the POP cohort. Compared to AOP patients, POP patients presented with a higher proportion of complex perianal fistulas (71.7% vs 50.0%, *p* = 0.011) and non-stricturing and non-penetrating (B1) behaviour (66.7% vs 47.8%, *p* = 0.005). Stricturing type (B2) behaviour (42.1% vs 27.4%, *p* = 0.024) and current smoking (12.9% vs 4.8%, *p* = 0.040) were more common among AOP patients than POP patients (Table 1).

**Table 1:** Clinical characteristics of PCD patients with paediatric-onset and adult-onset perianal disease

Factors	Patients with POP (N = 84)	Patients with AOP (N = 209)	*P *value
Male, n (%)	68 (81.0)	154 (73.7)	0.228
Mean age at CD diagnosis (years ± SD)	18.0 ± 4.3	32.0 ± 10.9	< 0.01
PCD diagnostic delay (months)	12 (2–48)	24 (2.5–60)	0.033
Disease location, n (%)			0.198
L1, terminal ileum	19 (22.6)	55 (26.3)	
L2, colon	17 (20.2)	62 (29.7)	
L3, ileocolon	45 (53.6)	87 (41.6)	
L4, upper gastrointestinal tract	3 (3.6)	5 (2.4)	
Disease behaviour, n (%)			0.015
B1, non-stricturing, non-penetrating	56 (66.7)	100 (47.8)	0.005^a^
B2, structuring	23 (27.4)	88 (42.1)	0.024^a^
B3, penetrating	5 (6.0)	21 (10.0)	0.364^a^
Mean PD onset age (years ± SD)	15.1 ± 2.9	30.2 ± 10.5	< 0.01
Perianal fistula, n (%)	53 (63.1)	110 (52.6)	0.119
Complex perianal fistula, n/N (%)	38/53 (71.7)	55/110 (50.0)	0.011
Perianal abscess, n (%)	41 (48.8)	100 (47.8)	0.898
Hemorrhoids, n (%)	16 (19.0)	35 (16.7)	0.639
Anal ulcer, n (%)	3 (3.6)	12 (5.7)	0.430
Anal fissures, n (%)	5 (6.0)	8 (3.8)	0.424
Rectovaginal fistula, n (%)	1 (1.2)	9 (4.3)	0.153
Rectal inflammation, n (%)	6 (7.1)	24 (11.5)	0.268
Current smokers, n (%)	4 (4.8)	27 (12.9)	0.040
Family history of IBD, n (%)	4 (4.8))	22 (10.5)	0.117
Medications, n (%)			
IFX	28 (33.3)	46 (22.0)	0.044
Immunomodulators	5 (6.0)	13 (6.2)	0.925
Antibiotics	51 (60.7)	128 (61.2)	0.933
Corticosteroids	3 (3.6)	7 (3.3)	1.000
Time from PCD diagnosis to first use of IFX, months (IQR)	1.0 (0.1–1.9)	1.8 (5.3–38.6)	< 0.01
Numbers of IFX uses (mean ± SD)	7.7 ± 0.75	7.1 ± 0.6	0.472
History of PSIs, n (%)	66 (78.6)	150 (71.8)	0.245
Frequency of PSIs, n (%)			0.284
1 time	46 (54.8)	118 (56.5)	
2 times	15 (17.9)	22 (10.5)	
3 times	5 (6.0)	10 (4.8)	
Types of perianal surgery, n (%)			
Incision and drainage	33 (39.3)	68 (32.5)	0.272
Seton insertion	42 (50.0)	83 (39.7)	0.107
Fistulectomy	19 (22.6)	52 (24.9)	0.764
Proctectomy	0 (0)	2 (1.0)	1.000
De-functioning	1 (1.2)	2 (1.0)	1.000
Procedure for prolapsing haemorrhoids	7 (8.3)	14 (6.7)	0.624
CD-related intestinal resection, n (%)	4 (4.8)	44 (21.1)	0.001
Indication for surgery, n (%)			0.815
Inflammation	0 (0)	3 (6.8)	
Obstruction	2 (50.0)	19 (43.2)	
Perforation	1 (25.0)	18 (40.9)	
Obstruction combined with perforation	1 (25.0)	4 (9.1)	
Extent of resected intestine, n (%)			0.232
Partial ileal resection	2 (50.0)	20 (45.5)	
Partial colon resection	1 (25.0)	2 (4.5)	
Ileocolonic resection	1 (25.0)	22 (50.0)	

*CD* Crohn’s disease, *PCD* perianal Crohn’s disease, *PD* perianal disease, *POP* paediatric-onset perianal disease, *AOP* adult-onset perianal disease, *SD* standard deviation, *IQR* interquartile range, *PSIs* perianal surgical interventions, *IFX* infliximab, *IBD* inflammatory bowel disease

^a^This was a subgroup analysis of three disease behaviours between the AOP and POP groups

A total of 253 patients received initial treatment for PD. Among them, PSIs combined with antibiotic treatment was performed on half of the PCD patients (153/293, 52.2%), then followed by IFX treatment after PSIs (36/293, 12.3%), IFX monotherapy (30/293, 10.2%), antibiotic monotherapy (26/293, 8.9%) and IFX combined with immunosuppressants (8/293, 2.7%). Two hundred forty-six PCD patients achieved clinical remission of PD after initial treatment, including 71 POP patients (84.5%) and 175 AOP patients (83.7%). Of the 246 patients who achieved clinical remission, 60 (24.4%) patients experienced PD recurrence during the follow-up period, including 21 (29.6%, 21/71) POP patients and 39 (22.3%, 39/175) AOP patients. The types of PD experiencing recurrence included 11 (52.4%) perianal abscess, 9 (42.9%) perianal fistulas, one (4.8) anal fissure, but no rectovaginal fistula in 21 POP patients. These frequencies in 39 AOP patients were 19 (48.7%), 19 (48.7%), 0 (0%) and 1 (2.6%), respectively (*p* = 0.609). No significant difference in the disease location, PSI frequency, PSI type or family history of IBD was observed between the two groups of patients (*p* > 0.05, Table 1).

### IFX treatment

Seventy-four patients (25.5%, 74/293) receiving IFX treatment for active PD (perianal fistulas, perianal abscess and rectovaginal fistulas) after the first diagnosis of PCD. Those PCD patients requiring IFX only for luminal disease were excluded. Among them, 30 patients received IFX monotherapy, 36 patients received IFX combined with PSI, and 8 patients were treated with both IFX and immunosuppressants. The average number of IFX infusions was 7.3 ± 0.5.

**Fig. 2:**
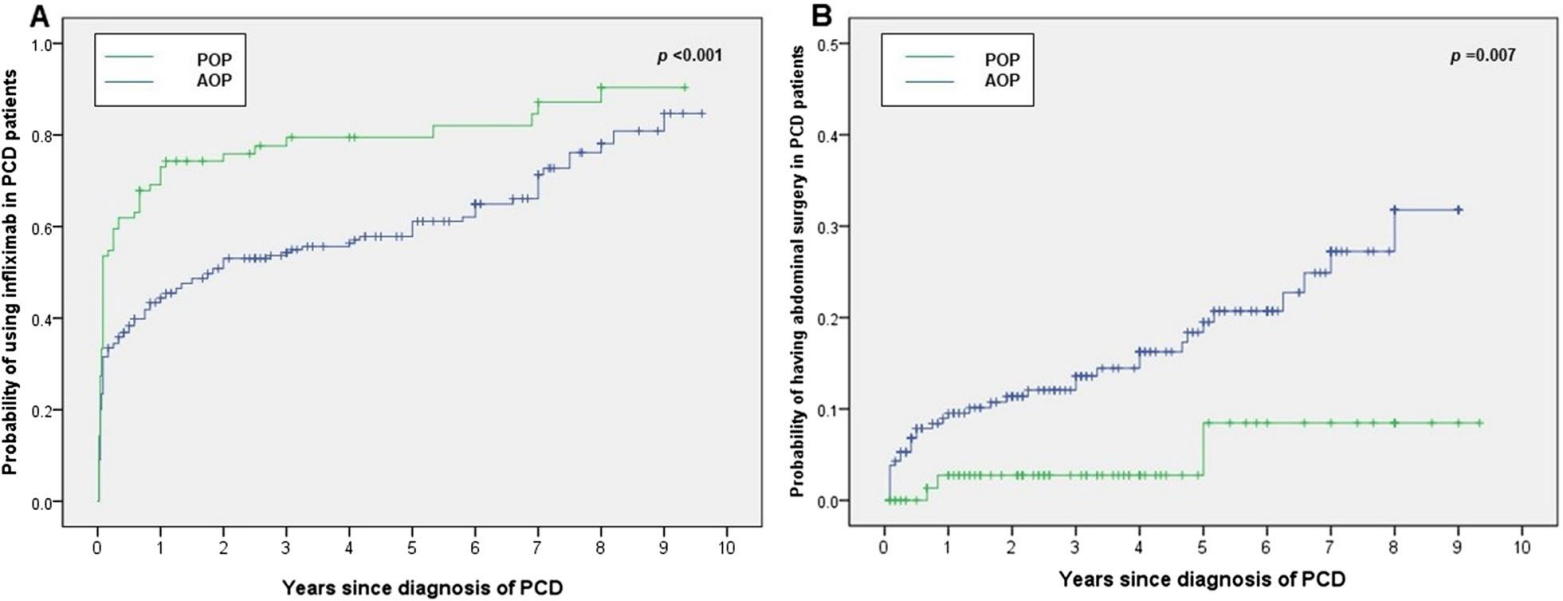
Cumulative probability of **a** infliximab and **b** CD-related intestinal resection in patients with paediatric-onset and adult-onset perianal disease. *PCD* perianal Crohn’s disease, *POP* paediatric-onset perianal disease, *AOP* adult-onset perianal disease

The proportion of patients treated with IFX was significantly higher in the POP group (33.3% vs 22.0%, *p* = 0.044, Table 1). The median time from diagnosis of PD to the first infusion of IFX was shorter among POP patients than AOP patients (1 month, IQR: 0.1–1.9 vs 22 months, IQR: 5.3–38.6, *p* < 0.01, Table 1). The cumulative probability of IFX therapy at 1, 3, 5 and 10 years was 69.1%, 77.6%, 79.5% and 90.4% in POP patients versus 43.4%, 53.7%, 57.8% and 84.7% in AOP patients, respectively (*p* < 0.001, Fig. 2A). No significant differences were detected at numbers of IFX infusion between patients with POP and AOP (*p* > 0.05, Table 1).

### Surgical treatment

Forty-eight patients (16.4%) suffered CD-related intestinal resection after the first diagnosis of PD. The percentage of patients who underwent surgery was significantly higher in the AOP cohort (21.1% vs 4.8%, *p* = 0.001, Table 1). However, there was no significant difference in the indication for surgery and the extent of resected intestine between POP and AOP patients (*p* > 0.05, Table 1). Furthermore, the cumulative probability of CD-related intestinal resection after CD diagnosis was also higher in AOP patients (9.0% at 1 year, 12.1% at 3 years, 18.4% at 5 years, and 31.8% at 10 years) than in POP patients (2.7% at 1 year, 2.7% at 3 years, 2.7% at 5 years, and 8.5% at 10 years, *p* = 0.007, Fig. 2B).

In the multivariate logistic analysis (Table 2), AOP (OR: 4.939, 95% CI 1.538–15.855, *p* = 0.007), stricturing behaviour (B2 vs B1, OR: 1.810, 95% CI 1.008–3.251, *p* = 0.047) and presence of rectal inflammation (OR: 3.166, 95% CI 1.119–8.959, *p* = 0.030) were associated with a higher risk of CD-related intestinal resection among the 293 PCD patients. Other factors, including sex, age at CD diagnosis, PCD diagnostic delay, current smoking status, PD type and disease location, were not associated with CD-related intestinal resection (*p* > 0.05). Furthermore, AOP patients with complex perianal fistulas were more likely to undergo CD-related intestinal resection (OR: 2.257, 95% CI 1.041–4.891, *p* = 0.039, Table 2). POP patients with rectal inflammation (OR: 7.000, 95% CI 1.876–15.407, *p* = 0.012) had increased risk for CD-related intestinal resection (Table 2).

**Table 2:** Factors associated with the risk of intestinal resection

Factors	Univariate analysis		Multivariate analysis	
	OR (95% CI)	*P*-value	OR (95% CI)	*P* value
*All PCD patients (n = 293)*				
Male	1.727 (0.883–3.379)	0.110	0.488 (0.194–1.225)	0.126
AOP	2.887 (1.511–5.515)	0.001	4.939 (1.538–15.855)	0.007
Behaviour		0.019		0.070
B2 versus B1	2.097 (1.194–3.685)	0.010	1.810 (1.008–3.251)	0.047
B3 versus B1	2.329 (0.833–6.513)	0.107	2.291 (0.789–6.651)	0.127
IFX + PSIs	0.270 (0.063–1.163)	0.079	0.243 (0.049–1.206)	0.083
Rectal inflammation	2.462 (1.050–5.769)	0.038	3.166 (1.119–8.959)	0.030
*Patients with AOP (n = 209)*				
PCD diagnostic delay	0.944 (0.986–1.001)	0.109	0.994 (0.986–1.001)	0.109
Behaviour		0.061		0.089
B1 versus B3	3.488 (0.435–27.976)	0.239	3.595 (0.440–29.390)	0.233
B2 versus B3	6.769 (0.858–53.416)	0.070	6.563 (0.817–52.714)	0.077
IFX + PSIs	0.324 (0.073–1.433)	0.138	0.374 (0.082–1.712)	0.205
Complex perianal fistulas	2.297 (1.089–4.848)	0.029	2.257 (1.041–4.891)	0.039
*Patients with POP (n = 84)*				
Family history of IBD	4.102 (0.861–19.532)	0.076	2.670 (0.273–26.148)	0.399
Rectal inflammation	9.000 (2.099–17.210)	0.009	7.000 (1.876–15.407)	0.012

*CD Crohn’s disease, *PD* perianal disease, *B1* non-stricturing and non-penetrating, *B2* stricturing, *B3* penetrating, *IFX* infliximab, *PSIs* perianal surgical interventions, *PCD* perianal Crohn’s disease, *AOP* adult-onset perianal disease, *POP* paediatric-onset perianal disease, *IBD* inflammatory bowel disease

### Trend of clinical characteristics in POP and AOP patients over the 10-year observation period

**Fig. 3:**
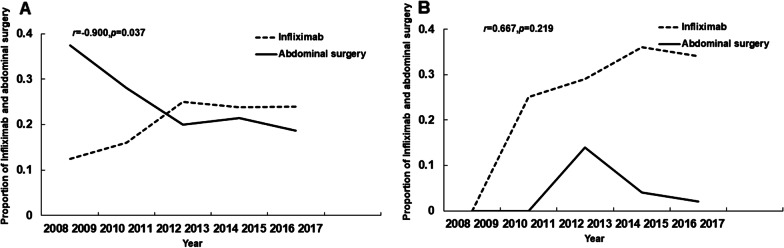
Ten-year trend and correlation between infliximab and CD-related intestinal resection in CD patients with **a** adult-onset and **b** paediatric-onset perianal disease

From 2008.1 to 2017.12, the proportion of patients receiving IFX treatment increased in both the POP and AOP groups (Fig. 3). The occurrence of CD-related intestinal resection showed a gradually decreasing trend in AOP patients (Fig. 3a). And a negative correlation was found between the proportion of patients receiving IFX treatment and undergoing intestinal resection only in AOP patients (*r* = − 0.900, *p* = 0.037) but not in POP patients (*r* = 0.667, *p* = 0.219).

## Discussion

The presence of PD in CD patients usually indicates increasing hospitalization, progression to more complicated disease phenotypes, and high rate of intestinal resection in some studies about Western patients [2, 23, 24]. The growing studies also reported that PD at the time of CD diagnosis was more common in Asian countries (33.7–42.4%) than in some Western countries (13.0–26.9%) [13, 19, 25, 26]. The increasing tendency in China over the past few decades calls for more attention to be paid to the natural course of PCD in these newly developed disease population. However, Chinese studies evaluating the characteristics and outcomes of PCD patients are limited, and the significance of PD development at different ages is unclear. This is the first comparative study to investigate this issue among a Chinese 10-year observational cohort study in the first years of the biologic treatment era. Thus, this study allowed us to obtain key updated knowledge about the differences between PCD patients with paediatric and adult‐onset PD concerning the need for intestinal resection. Firstly, we found that CD patients who developed PD during childhood had a more serious perianal phenotype and earlier PCD diagnosis and IFX treatment, while aggressive luminal disease, current smoking and a higher rate of intestinal resection were more common in AOP patients. Secondly, AOP, stricturing luminal behaviour and rectal inflammation were associated with an increased incidence of intestinal resection. And AOP patients with complex perianal fistulas or POP patients with rectal inflammation were more likely to undergo CD-related intestinal resection. Finally, IFX administration could decrease the rate of intestinal resection in AOP patients. These results indicate that more attention should be paid to AOP patients with complex perianal fistulas and POP patients with rectal inflammation, and highlight the important role of early diagnosis and IFX therapy in achieving better clinical outcomes.

Previous studies have shown that approximately one-quarter of CD patients are diagnosed in childhood [16]. Paediatric CD patients usually show distinct features, which are characterized by more severe disease activity and biological therapy than adult CD cases [17, 27]. In our study, 28.7% of PCD patients developed their first perianal symptoms during the paediatric period, and they presented with more complex perianal fistulas. However, the stricturing phenotype of luminal CD and CD-related intestinal resection was more common in AOP patients. Thus, early-onset (paediatric-onset) PD presents more complicated local manifestations of PD, but late-onset (adult-onset) PD is more prone to progress to severe luminal damage progression. These results not only indicate distinct clinical characteristics and monitoring focus in CD patients who develop PD at different ages but also suggest the presence of distinct disease patterns for perianal and luminal CD.

Moreover, we found that PCD patients with POP had earlier use of IFX and a higher cumulative risk of IFX treatment. In the past decade, IFX was the only available biological agent for Chinese CD patients [28]. Due to its efficacy in treating complicated CD, IFX has been recommended widespread in cases of early-onset or fistulizing PCD as a top-down treatment strategy in recent years [4–10]. We found that POP has been increasing over time, which means there is a trend for POP patients to be diagnosed and treated more recently. PCD diagnosis was performed earlier in POP patients than AOP patients. Therefore, the higher percentage of complex perianal fistulas, early-onset and recent diagnosis of PD in the POP group may contribute to the more frequent and earlier use of IFX.

The presence of PD speeds up the complexity of the natural history of CD, accelerates the development of luminal stenosis, and increases the risk of intestinal resection in some studies of the pre-biologic era [2, 23, 24]. However, most of these studies focused on the adult-onset cohort, they neglected the influence of age of the PD development and biological agents on the CD outcomes. Recently, a Korean study reported that the presence of PD at CD diagnosis did not increase the risk for abdominal surgical intervention [13]. And a Danish population-based study found that the occurrence of PD at CD diagnosis was not predictive of the risk for undergoing intestinal resection, whereas the development of PD during follow-up was [1]. These results raise the possibility that the impact of the PD development time on the intestinal resection of CD patients may differ. The later the PD developed, the more common the complicated luminal damage and intestinal resection presented. This result is inconsistent with the previous conclusion that the early-onset CD was associated with an increased incidence of CD-related surgery [29]. But our results are similar to a recent study. They showed that there were more intestinal strictures and abdominal operations in adult CD patients than in paediatric CD patients, which was related to the longer diagnostic delay in adults [18]. Similarly, we found the patient with AOP (late-onset PD) presented a higher rate of stricturing behaviour and intestinal resection. Factors including AOP, stricturing behaviour and rectal inflammation were predictive for increased CD-related intestinal resection among all PCD patients. The use of IFX treatment also showed a negative correlation with the rate of intestinal resection in patients with AOP. This verified the conclusion that the increased use of biologics is positively correlated with a reduced need for intestinal resection [30]. Furthermore, we observed that the time from the onset of PD to the diagnosis of PCD and the time from the diagnosis of PCD to the first use of IFX were longer in the adult-onset cohort. These results all suggest a later disease course and later IFX treatment in AOP patients. It has become increasingly apparent that the early use of IFX can decrease the occurrence of CD-related surgery in adult CD [19, 31]. Therefore, the more complicated luminal disease, underuse of IFX treatment and delayed initiation of IFX may contribute to the higher rate of intestinal resection in AOP patients.

In addition, smoking has been reported as a definite factor associated with recurrence after surgery and a poor response to medical therapy in CD patients [32]. Thus, the greater proportion of current smokers also explained the higher rate of intestinal resection in AOP patients. Unexpectedly, both in the univariate and multivariate logistic analysis, we didn’t find that current smoking status was significantly associated with CD-related abdominal surgery. This result is inconsistent with previous conclusion. The reason may contribute to a very small samples (n = 4) of current smokers in POP patients, and this can lead to the bias in the result. Taken together, these findings indicate that a close monitoring of luminal lesions and a positive therapeutic strategy, including the early and extensive use of IFX treatment and cessation of smoking, should be undertaken in PCD patients with AOP to reduce the rate of intestinal resection.

One of the strengths of our study is the inclusion of all kinds of PCD, leading to a reduction in selection bias. The different clinical characteristics and outcomes of PCD patients with POP and AOP were directly compared in this study. We focused on the influence of PD onset age on the rate of intestinal resection in CD patients, and found that there was a higher risk of intestinal resection in PCD patients who developed PD at adults. These findings should alert physicians to pay close attention to these patients to ensure that they receive intensive treatment and close luminal monitoring to achieve better outcomes in clinical practice.

However, there are some limitations to our study. The first limitation is the retrospective and single-centre design. Secondly, all of the PCD patients included in our study were hospitalized patients, who may have had relatively serious manifestations. Lastly, some CD patients developed PDs in an early time without more detailed records of PSIs. We failed to provide a more specific description of PSIs. So, we can’t analyse the influence of different PSIs types on the perianal outcome, which was still needed to be further studied and confirmed.

In summary, POP and AOP play different roles in pathogenesis and progression of CD. POP showed more complicated perianal lesions than AOP. The clinical impact of PD on the luminal damage of CD patients may be more prominent in patients with AOP than those with POP. Early diagnosis and IFX treatment, as well as closely endoscopic evaluation in CD patients with AOP may therefore allow for earlier identification of potential CD-related luminal damage to reduce the incidence of intestinal resection.

## Data Availability

The datasets used and analysed during the current study available from the corresponding author on reasonable request.

## Abbreviations

CD: Crohn’s disease
PD: Perianal disease
PCD: Perianal Crohn’s disease
IBD: Inflammatory bowel disease
POP: Paediatric-onset PD
AOP: Adult-onset PD
TNF: Tumour necrosis factor
IFX: Infliximab
PSIs: Perianal surgical interventions
